# A Two-Dimensional Wireless and Passive Sensor for Stress Monitoring

**DOI:** 10.3390/s19010135

**Published:** 2019-01-02

**Authors:** Yisong Tan, Jianhua Zhu, Limin Ren

**Affiliations:** School of Mechanical Engineering, Northeast Electric Power University, Jilin 132012, China; tanyisong@neepu.edu.cn (Y.T.); 2201600247@neepu.edu.cn (J.Z.)

**Keywords:** passive and wireless sensor, stress monitoring, magnetic material, smart structure, finite element analysis

## Abstract

A new two-dimensional wireless and passive stress sensor using the inverse magnetostrictive effect is proposed, designed, analyzed, fabricated, and tested in this work. Three pieces of magnetostrictive material are bonded on the surface of a smart elastomer structure base to form the sensor. Using the external load, an amplitude change in the higher-order harmonic signal of the magnetic material is detected (as a result of the passive variation of the magnetic permeability wirelessly). The finite element method (FEM) is used to accomplish the design and analysis process. The strain-sensitive regions of the tension and torque are distributed at different locations, following the FEM analysis. After the fabrication of a sensor prototype, the mechanical output performance is measured. The effective measurement range is 0–40 N and 0–4 N·M under tension and torque, respectively. Finally, the error of the sensor after calibration and decoupling for F_x_ is 3.4% and for T_x_ is 4.2% under a compound test load (35 N and 3.5 N·M). The proposed sensor exhibits the merits of being passive and wireless, and has an ingenious structure. This passive and wireless sensor is useful for the long-term detection of mechanical loading within a moving object, and can even potentially be used for tracing dangerous overloads and for preventing implant failures by monitoring the deformation of implants in the human body.

## 1. Introduction

Strain sensors are often used to detect physical deformation and mechanical loads, such as stress and strain, on a structure. To measure strain and stress quantitatively, these sensors are normally installed at a position that is strain-sensitive, and they communicate with an external instrument to obtain an output signal. Stress and strain monitoring are important for studying the structural condition and for providing critical information for further structural design and improvement. Nowadays, strain sensors are used in structure health monitoring (SHM), such as civil constructions and clinical medical applications [1,2].

In civil constructions, the use of a strain sensor for SHM was demonstrated by Ausanio [3], who monitored the structural condition using a magnetoelastic sensor. Similarly, Ansari [4] demonstrated the monitoring of structural health using a long gage and fiber optic acoustic sensors. The long-term monitoring of strain or stress within sensitive structures, such as roadways, bridges, and building supports, is important in order to ensure that their mechanical properties are within the safety limits [5]. SHM has often reduced the unnecessary replacement of parts. Moreover, strain or stress monitoring provides a method to estimate the fatigue of a structure, thus preventing structural failure [6].

In medical applications, F. Umbrecht [7] developed a wireless and passive implantable sensor based on ultrasound detection for the real-time monitoring of small strain on implants, bones, and joints. Another real-time strain sensor is the magnetostrictive amorphous sensor, presented by T. Klinger [8], which measures strain by detecting the changes in the magnetic permeability caused by mechanical loading. However, the above methods are only for single-dimension force detection and cannot detect a two-dimensional force. Therefore, special attention should be paid to the mechanical testing of the composite loads of tension and torque.

While there are many types of two-dimensional and even multidimensional sensors, the most common and established ones are the resistive and capacitive strain gauges. For example, the vertical beam six-axis force sensor, developed by Waston [9] of the Draper Research Institute, is based on the change of resistance value caused by the deformation of the resistor strain gauge under an external load, and the force or torque is converted into an electric signal. A capacitive six-axis force sensor was also designed by Givanazzo [10]. There are many groups of electrodes on the elastomer of the sensor. When a force acts on the elastomer, the electrode moves with the elastomer, which makes the capacitance of each dimension change in direct proportion to the force. The above-mentioned resistor and capacitive strain sensors, which are used extensively for damage monitoring, are simple and sensitive. However, they do present certain limitations. Most of the existing sensors need to be connected directly to electronic circuits in order to transmit data and power, making them unsuitable for monitoring the real-time stress within a structure for a long period of time [11].

For these reasons, we propose a two-dimensional wireless and passive sensor for stress monitoring for the first time. The sensor signal is detected and evaluated from outside the structure, using a magnetic field. The proposed sensor uses the magnetostrictive material as the underlying sensing element, which can be used in a wireless passive configuration to monitor the two-dimensional force in real-time and long-term. In this paper, the working principle of the magnetostrictive material is discussed, then, the design, analysis, and fabrication processes of the sensor are described. Finally, a wireless and passive sensor prototype is fabricated, and its mechanical output characteristics are tested. Furthermore, the proposed sensor is compared with two existing implantable strain sensors. This two-dimensional wireless and passive sensor can potentially be used for monitoring the stress within a structure.

## 2. Materials and Methods

### 2.1. Materials and Principle

**Figure 1:**
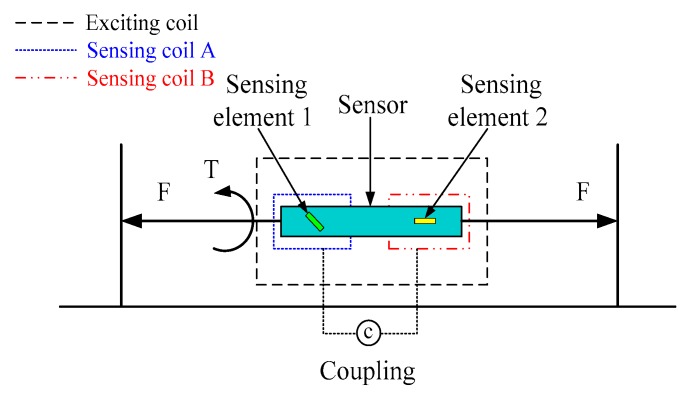
A schematic diagram of the operating principle.

The working principle of the force sensor is based on the inverse magnetostrictive effect, which measures stress by detecting a change in the magnetic permeability, caused by the mechanical load, in a wireless and passive manner. The sensing material is Fe_40_Ni_38_Mo_4_B_18_ (model 2826 MB, Metglas Inc., Hong Kong, China), which is a ribbon-like amorphous ferromagnetic alloy. The biocompatible application of the magnetostrictive material has existed for many years [12,13,14,15]. The Fe_40_Ni_38_Mo_4_B_18_ alloy not only exhibits a high permeability, low resistivity, high magnetostriction coefficient, and high mechanical tensile strength, but has a low material cost, which allows for it to be used on a disposable basis [16,17,18]. In addition, the material has a high magnetoelastic coupling coefficient, as high as 0.98, and a magnetostriction to the order of 10^−5^, which allows for efficient conversion between magnetic and elastic energies, and vice versa [19,20]. The operating principle representation is shown in Figure 1. The sensor is in the magnetic field, which is generated by an exciting coil. When the sensor is subjected to tension or torque strain, the permeability of the Fe_40_Ni_38_Mo_4_B_18_ alloy will change accordingly with the force. At the same time, the permeability variation leads to the change of magnetic flux density, which is obtained by sensing coils A and B, respectively.

### 2.2. Sensor Design, Analysis, and Fabrication

**Figure 2:**
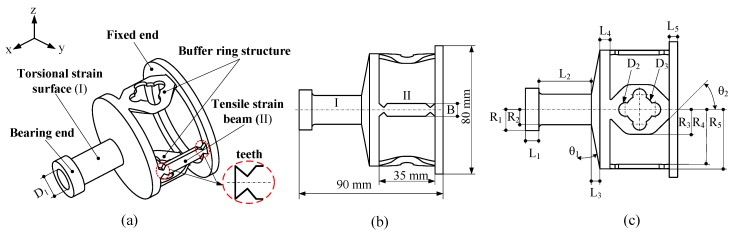
The structure of the sensor: (**a**) three-dimensional model, (**b**) main structure dimension, and (**c**) specific structure dimensions.

The sensor is applied in order to detect two-dimensional force information. The coupling between the force and torque measurement is inevitable. The main factor influencing the coupling is the structure of the sensor itself, so it is very important to design the elastomer structure of the sensor. A geometric model of the sensor is created using the three-dimensional (3D) software Pro/Engineer. Figure 2 shows the three-dimensional model and the structure dimensions of the sensor, in which the strain-sensitive regions of tension and torque are marked by areas “I” and “II”. The proposed sensor mainly consists of the bearing end, tensile strain beam, torsional strain surface, buffer ring, and fixed end.

The tensile strain beam is a strip-type, with a symmetrical teeth distribution at both ends. The torsional strain surface is a central symmetric hollow cylindrical thin-walled structure. The tensile strain beams and torsional strain surface are bonded with sensing materials in order to detect the stress caused by tension and torque, respectively. The fixed end and bearing end are used for the sensor’s installation, fixation, and loading application, respectively. The buffer ring structure is a thin-walled structure formed by arranging some holes through the surface of the polygon. The principle of the structure decoupling method is based on the stiffness distribution. Through the design of the smart structure, the torque- and tension-sensitive regions are distributed more reasonably under the action of the buffer transition. Figure 2 and Table 1 both show the specific structure dimensions of the sensor.

**Figure 3:**
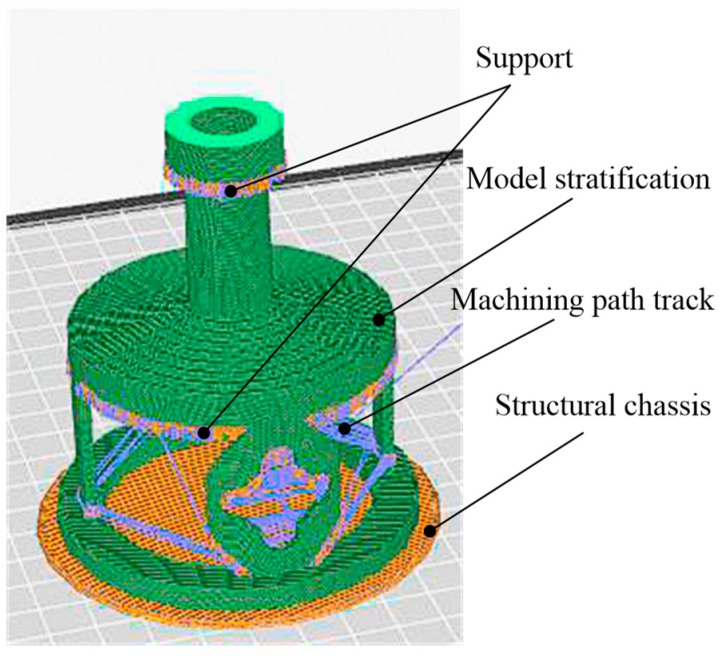
Stratification of the sensor before three-dimensional (3D) printing.

If this structure adopts the form of a split combination, there will be some problems in the design and manufacture of the mechanical structure, such as the matching clearance and dead zone. Therefore, an elastic integrated structure is adopted; however, the overall manufacture difficulty is greatly increased. This integral hollow thin-walled structure is difficult to achieve using the traditional mechanical processing method. Our design benefits from 3D printing technology, which is different from the traditional processing technology of removing the material. The three-dimensional entity is generated by adding the material layer-by-layer. Figure 3 is the stratification of the structure before 3D printing. According to the requirements of precision, as well as other relevant parameters, the model is cut in a parallel plane along the forming direction of the part. The orange bottom is the structure chassis for the entire 3D printing, the green part is the stratification effect of the three-dimensional entity, and the intersection of the purple and yellow lines represents the support and path in the stacking process. Finally, the model will be imported into the 3D printer, and the machining path will be formed according to the stratification. The entity model will be processed and accumulated layer-by-layer until the model is completed. All parts of the sensor, except for the sensing elements, are manufactured by the MakerBot Replicator Z183D (LLC One Metro Tech Center, Brooklyn, NY, USA) printer. The material of the elastomer structure is polylactic acid (PLA), which is a biocompatible [21,22,23] thermosensitive plastic extracted from corn starch.

**Figure 4:**
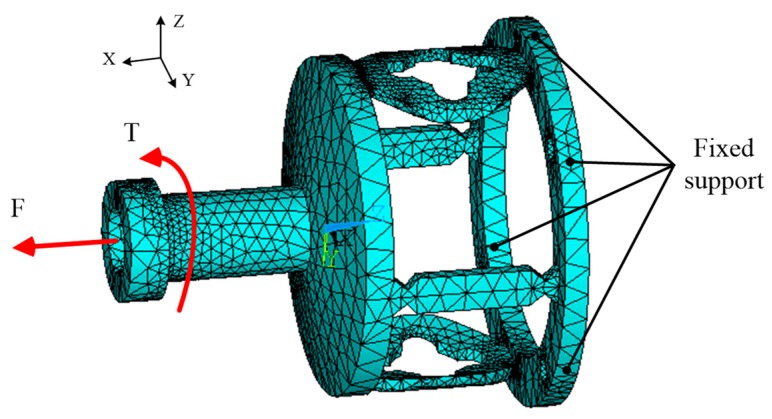
The finite element model of the elastomer.

In order to obtain the optimal strain-sensitive regions of the tension and torque of the sensor, the finite element method (FEM) is used in this paper to accomplish the design and analysis. According to the structural parameters of the sensor listed in Table 1, the finite element (FE) model of the sensor is created, and the SOLID187 element is adopted for meshing using the ANSYS software. Table 2 shows the number of elements and nodes used in the FE model. The material properties used for the sensor structure are listed in Table 2. FEM is used for analyzing the strain-sensitive regions when the sensor is under load. In order to analyze the strain of the sensor under external loads, the loads and constraint conditions are set in the established model. The results of the model meshing and force constraint conditions are shown in Figure 4.

**Figure 5:**
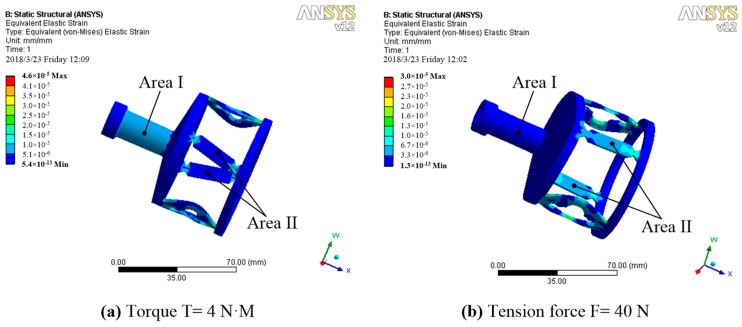
Strain distribution diagrams under different forces: (**a**) strain distribution at T = 4 N·M and (**b**) strain distribution at F = 40 N.

The static structure simulation of the 3D elastomer structure model is accomplished using ANSYS. The corresponding tensile-sensitive region and the torque-sensitive region are obtained using an FEM analysis. The results of the analysis are shown in Figure 5a,b. When the elastomer structure is subjected to the torque of T = 4 N·M alone, the whole structure is subjected to torsional shear stress. The torsional area I can withstand remarkable strain. The buffer ring bears most of the torque effect. Only a small shear force acts on the tensile area II, and the shear effect is concentrated at the two ends of the tensile beam. The main part of the tensile beam bears very little torque strain. Finally, the strain in torsional region I, due to the torque, is higher than that of region II. When the elastomer structure is subjected to the tension force of F = 40 N alone, the torsional strain surface can transfer most of the tension force to the tensile strain beam in the case of very small strain, owing to its high tensile stiffness. The tensile strain beam has an obvious strain because of its tension sensitiveness. Therefore, the strain sensitivity of strain region II to the tensile force is much greater than that of region I.

**Figure 6:**
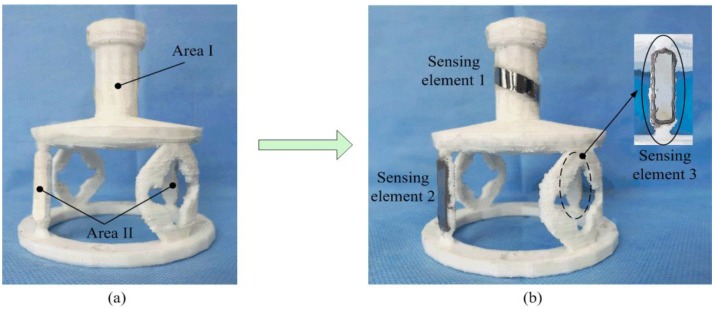
The prototype of the proposed sensor: (**a**) the elastomer structure and (**b**) the position of the three sensing elements.

The elastomer structure of the two-dimensional sensor proposed in this paper is based on the stiffness distribution. Through the reasonable distribution of stiffness from the sensor structure, the sensitive areas under tension and torque effects are at different positions, which can reduce the dimensional coupling to the largest extent. Finally, according to the results of the strain analysis and the mechanical characteristics of the materials, three pieces of the ribbon-like magnetostrictive sensing elements are stuck on the surface of the elastomer structure, as shown in Figure 6. The sensing elements of the sensor in this study have a size of 25 mm × 7 mm × 28 μm (length × width × thickness). The magnetostrictive sensing element of torque-sensitive region I is arranged at an angle of 45° to the central axis. The magnetostrictive sensing elements of tensile-sensitive region II are two parallel, diametrically opposite pieces. A high viscosity adhesive is selected for the sensor, so as to guarantee a rigid state. A high stiffness adhesive, J-B Weld^TM^ (Northeast Texas, TX, USA), is adopted in the process of the sensor’s fabrication [24].

### 2.3. Sensor Output Performance Experiments

**Figure 7:**
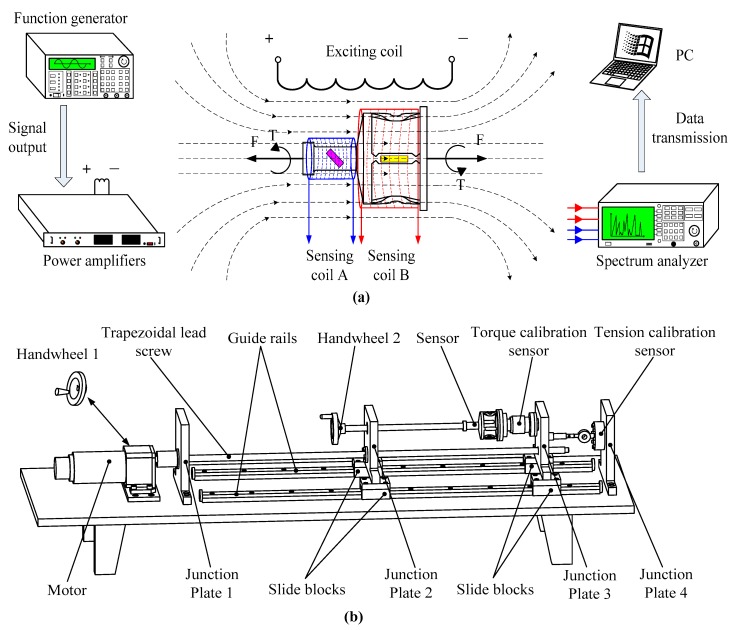
The experiment device schematic diagram: (**a**) method for the output performance experiments and (**b**) the schematic diagram of the loading platform. PC, personal computer.

Figure 7a shows a sketch diagram of the experiment setup for the output performance measurement. The stress of the sensor could be detected via the sensing coils. In order to analyze the tension and torque variations of the sensor, the experiment devices need to be equipped with accurate tension and torque sensors, as well as digital display instruments. Hence, a two-dimensional loading platform is developed in this paper. The two-dimensional loading platform consists of guide rails, sliding blocks, a trapezoidal lead screw, junction plates, handwheels, and display instruments, which are used for calibrating the tension and torque sensors. As shown in Figure 7b, the two-dimensional sensor is installed on the testing platform for force loading. The loading platform is placed on the guide rails, with high-linear precision. This not only reduces the frictional resistance while applying as much force as possible, but it also ensures the high accuracy of the experiment. The sensor is fixed between two junction plates using a bolted connection. The junction plates are fixed on the sliding blocks, which can move along the parallel guide rails. The platform could apply tension and torque to the sensor through two handwheels connected to the left end.

**Figure 8:**
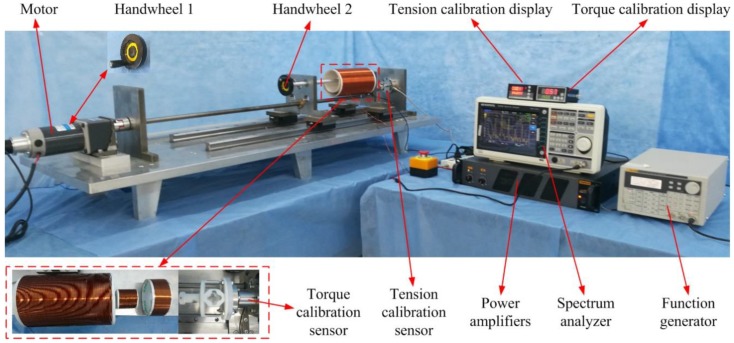
The experiment setup.

The detection system includes one exciting coil and two sensing coils in order to detect the magnetic permeability variation when the sensor is loaded. The specific parameters of the three coils are shown in Table 3. During the experiment, an alternating magnetic field is generated under the excitation of the external sinusoidal signal, which is given by a function generator. The sensor suffers from strains in the alternating magnetic field and generates the inverse magnetostriction effect, which results in magnetic flux variation in the two exciting coils. The changing signals are acquired by the sensing coils linked to a spectrum analyzer. Finally, the analysis data are transmitted and stored in the computer. The experiment setup is as shown in Figure 8.

## 3. Results and Discussion

### 3.1. Experimental Results of the Sensor Prototype

The output performance of the sensor has been tested in the experiments. The DDS Function Generator (Fluke 271, Fluke Corporation, Everett, WA, USA) was used for generating the sinusoidal signal, with a frequency of 200 Hz and a voltage of 2 V (peak to peak). The sinusoidal signal was amplified by the Power Amplifier (TAPCO Juice^TM^, LOUD Technologies Inc., Washington, DC, USA), and was input into the exciting coil in order to produce an alternating magnetic field (150 A/m). External forces were applied to the sensor using the two-dimensional loading platform. When the tension force or torque was loaded, the elastomer structure of the sensor was strained as a result of elastic deformation, and the magnetic permeability of the magnetostrictive material changed accordingly. The magnetic permeability variation could be detected by sensing coils A and B. Finally, the analysis results were displayed via the Spectrum Analyzers (GA40XX, Guorui Antai Technology Co. LTD., Shenzhen, China).

In order to collect the optimal experimental data, we marked the peak values of the first-, second-, and third-order harmonic signals successively. It was found that the third-order harmonic signal had an obvious variation and a more stable performance in this experiment. Therefore, the experimental data under the frequency of the third-order harmonic signals were collected.

**Figure 9:**
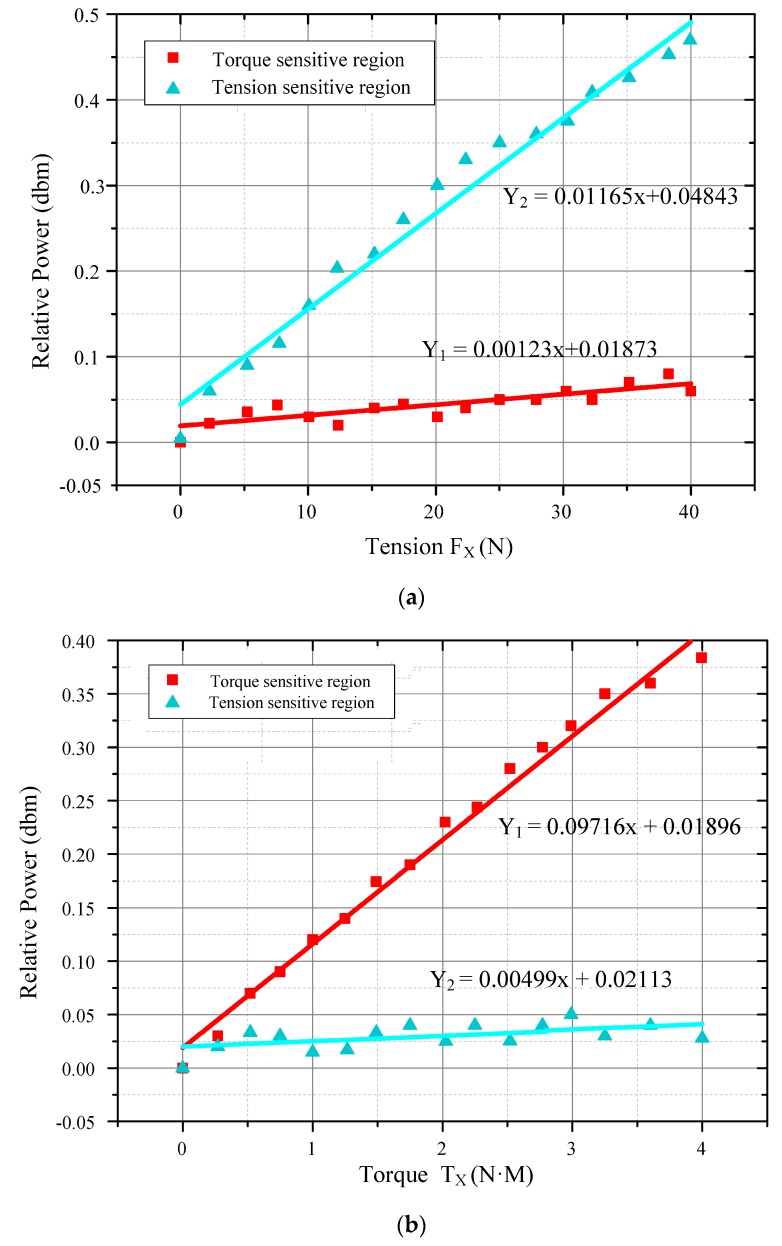
The output performance curve and linear fitting of the sensor: (**a**) output performance curve under tension and (**b**) output performance curve under torsion torque.

Figure 9a,b shows the relationships between the external force and the relative power of the detected third-order harmonic signals. In Figure 9a, a separate tension force F_x_ was applied to the sensor through the platform. Responses to the force F_x_ arose in the torque sensitive region I and force sensitive region II at the same time. With the F_x_ increasing, the response increased also. The relationship between the force F_x_ and the response in force-sensitive region II was almost a linear one. The linear fitting result was Y_2_ = 0.01165x + 0.04843 and the slope was 0.01165. In the torque-sensitive region I, a response to the force F_x_ also existed, but the varying tendency was much more different. Under the same external force F_x_, only a very small output was detected by the sensing coil A. The linear fitting result was Y_1_ = 0.00123x + 0.01873 and the slope was 0.00123. The two different slopes indicated that the force-sensing region was much more sensitive than the torque-sensing region to the external tension force. In Figure 9b, the same situation happened when applying a separate torque T_x_. A much larger increase was detected in the torque-sensitive region than in the force-sensitive region. The relationship between the torque input and response of the sensor is Y_1_ = 0.09716x + 0.01896 in the torque-sensitive region and Y_2_ = 0.00499x + 0.02113 in the tension-sensitive region. Finally, the measurement range of the tension and torque was 0–40 N and 0–4 N·M, respectively. After that, the output of the sensing coils did not change with the external force, as the magnetostriction materials gradually became saturated (the saturation magnetostriction of the sensor was 12 ppm from the datasheet of Metglas Inc., Conway, SC, USA).

### 3.2. Discussion

From the output curve of the tension F_x_ in Figure 9, it can be seen that an additional equivalent torque T_FX_ will be produced. From the output curve of the torque T_x_, it can be seen that an additional equivalent force F_TX_ will also be produced when loading the torque T_x_. However, these additional ones (T_FX_, F_TX_) are much smaller than their corresponding main ones (F_x_, T_x_). The experimental results of the mechanical output performance agree well with the static structure simulation of the model using the FEM analysis.

From Figure 9, it can be concluded that only low dimensional coupling exists in the sensor, owing to a reasonable smart structure as well as to the flexible arrangement of the sensitive materials. The decoupling operation will be done more for practical purposes. The traditional static decoupling method is based on the static linear calibration experiment. Assuming that the sensor is a linear measurement system with an input and output, the calibration matrix is determined using the experimental calibration of the sensor in all directions. The linear fitting of the data and its linear formulas are shown in Figure 9.

The applied load can be represented by the force matrix of F, and the output data can be expressed in the matrix of U. Then, the quantization relationship between the input matrix and the output value matrix can be expressed as follows:

(1) $$F=\left[\begin{array}{cc} F_{x} & 0\\ 0 & T_{x} \end{array}\right]\;U=\left[\begin{array}{cc} U_{11} & U_{12}\\ U_{21} & U_{22} \end{array}\right]\;F=\left[\begin{array}{cc} C_{11} & C_{12}\\ C_{21} & C_{22} \end{array}\right]\cdot U$$

It can be abbreviated as follows:

F = C·U(2)

In Formula (2), C is the calibration matrix, which is also called the coupling matrix, because it can reflect the coupling relationship between the forces of each of the dimensions. Hence, the coupling matrix, C, can be obtained using Formula (3). The function relation is as follows:

C = F·U^−1^ (3)

C is obtained and shown in Formula (4), as follows:

(4) $$C=\left[\begin{array}{cc} 79.0960 & -14.8305\\ -0.9887 & 11.1229 \end{array}\right]$$

From the calibration matrix, C, it can be concluded that the coupling component is very small. Especially in the second row of the matrix, the proportion of the coupling component and the main component is 1:11.2 (0.9887/11.1229). This indicates that the structure proposed in the paper has a certain ability for self-decoupling.

In order to obtain the actual performance of the sensor, a composite force loading experiment was carried out on the sensor. A compound test force (35 N) and torque (3.5 N·M) were applied to the sensor at the same time, and the output data from the two sensing coils were obtained. Then, by using the calibration matrix, C, and the sensor output data, U′, which were collected in the composite force loading experiment, the two-dimensional force matrix, F′, could be obtained by introducing it into Formula (2).

(5) $$U^{\prime }=\left[\begin{array}{cc} 0.46 & 0\\ 0 & 0.33 \end{array}\right]\;F^{\prime }=\left[\begin{array}{cc} 36.3842 & -5.1907\\ -0.4548 & 3.6706 \end{array}\right]$$

(6) $$\Delta =\left|\frac{F^{\prime }-F}{F_{\max}}\right|\times 100\%$$

After the linear calibration decoupling, we compared the results obtained to the F′ = (36.3842, 3.6706) and the input force matrix F = (35, 3.5); the errors of the two-dimensional wireless passive sensor can be calculated using Formula (6). Finally, the errors of the sensor were obtained as follows: F_x_ is 3.4% and T_x_ is 4.2%.

The bandwidth problem of the sensor was also studied. After numerous experiments, the bandwidth of the sensor was determined to be 47 Hz, which is much lower than the interrogation frequency (600 Hz). That is also to say the sweeping time of the spectrum analyzer is the main factor that limits the bandwidth of the whole system.

The key concern of this paper is monitoring the two-dimensional stress of the sensor in a wireless and passive way. A novel smart structure of the sensor plays an important role in its design, analysis, and fabrication. The most important part of the sensor is the selection of sensitive materials. The magnetostrictive material makes the sensor work wirelessly and passively; therefore, it can be implanted within the body to potentially monitor stress over a long period of time. The elastomer smart structure of the sensor ensures the relatively high accuracy of the output performance.

A qualitative comparison between the proposed sensor in this paper and the existing implantable strain sensors is discussed. This novel sensor is similar to the implantable sensors proposed by F. Alfaro [25] and F. Umbrecht [26], respectively, in bone stress monitoring. Both of them detect stress in a wireless manner. However, the actuating mechanisms of the three sensors are different. F. Alfaro’s sensor used a wireless piezoresistive bone stress sensor with an active electronics readout, which comprises an array of piezoresistive pixels to detect a stress tensor at the interfacial area between the Microelectro Mechanical Systems (MEMS) chip and the bone. However, the MEMS chip needs lead wires to supply power. The F. Umbrecht’s measurement principle is based on the transformation of an external force into a varying amount of fluid in a microchannel integrated into the sensor. The ultrasound read-out method based on an integral evaluation of the C-scans of the microchannel is proposed so as to determine the amount of fluid in the microchannel. However, only one-dimensional force information can be detected by it. The novel sensor proposed in this paper is based on the inverse magnetostrictive effect, and only uses the signals of the third-order harmonic frequency to monitor the stress information of the force sensor, which can not only detect two-dimensional force information, but also use a wireless and passive method at the same time.

## 4. Conclusions

A two-dimensional wireless and passive sensor for stress monitoring is proposed, designed, analyzed, fabricated, and tested for the first time. Three pieces of magnetostrictive material are bonded on an elastomer smart structure base to form the sensor. The measurement principle is based on the inverse magnetostrictive effect of the magnetostrictive amorphous magnetic ribbons. The variation of the stress is reflected by detecting the change in the magnetic permeability caused by mechanical loading. The smart structure of the sensor is based on the stiffness distribution, which makes the sensor have a particular decoupling ability. The decoupling performance of the structure is verified. The measured experimental results of the mechanical output performance agree well with the FEM analysis. The sensor prototype achieves an effective measurement range of 0–40 N for the tension force, and a 0–4 N·M for the torque. Finally, the error of the sensor after calibration and decoupling for F_x_ is 3.4% and for T_x_ is 4.2%. Compared with the detection methods in the medical fields, there is no physical connection between the sensor and the detector. Therefore, it can potentially be implanted in vivo to monitor the force information of bones and joints in order to prevent failure due to overloading. Furthermore, it has the ability to detect the two-dimensional sensor force information at the same time, which is significant for the mechanical detection of orthopedic implants. Future work will focus on the improvement of the mechanical output performance, the miniaturization of the structure, and the application in implantable medical sensors.

## Figures and Tables

**Table 1:** Specific structure dimensions.

Structure Dimensions	Values	Structure Dimensions	Values
D_1_	14 mm	B	8 mm
D_2_	8 mm	R_1_	12.5 mm
D_3_	16 mm	R_2_	9 mm
L_1_	8 mm	R_3_	20 mm
L_2_	30 mm	R_4_	32.5 mm
L_3_	5 mm	R_5_	35 mm
L_4_	6 mm	θ_1_	20°
L_5_	5 mm	θ_2_	45°

**Table 2:** Details of the finite element (FE) model and of the material properties used in the finite element method (FEM).

FEM Model	Elements/Nodes	Material Properties		
		Young′s Modulus	Tensile Strength	Poisson’s Ratio
Elastomer	3060/6343	1.7–3 (GPa)	≥60 (MPa)	0.36

**Table 3:** Specific parameters of the three coils.

Coil	Material	Diameter	Turns	Copper Diameter	Number of Layers
Exciting coil	Copper wire	110 mm	200	0.5 mm	Single
Sensing coil A	Single	40 mm	160	0.25 mm	Double
Sensing coil B	Single	80 mm	160	0.25 mm	Double
